# Assessment of Public Hospital Governance in Romania: Lessons From 10 Case Studies

**DOI:** 10.15171/ijhpm.2018.120

**Published:** 2018-12-22

**Authors:** Antonio Duran, Tata Chanturidze, Adrian Gheorghe, Antonio Moreno

**Affiliations:** ^1^ALLDMHEALTH, Seville, Spain.; ^2^Oxford Policy Management, Oxford, UK.

**Keywords:** Hospital Performance Assessment, Governance Framework, Romania

## Abstract

**Background:** The Government of Romania commissioned international technical assistance to help unpacking the causes of arrears in selected public hospitals. Emphases were placed on the governance-related determinants of the hospital performance in the context of the Romanian health system.

**Methods:** The assessment was structured around a public hospital governance framework examining 4 dimensions: institutional arrangements, financing arrangements, accountability arrangements and correspondence between responsibility and decision-making capacity. The framework was operationalized using a 2-pronged approach: (i) a policy review of broader health system governance arrangements influencing hospital performance; and (ii) a series of 10 casestudies of public hospitals experiencing financial hardship. Data were collected during 2016-2017 through key informant interviews with central authorities and hospital management teams, exhaustive semi-structured questionnaires filled in by hospitals, as well as the review of documentary sources where feasible.

**Results:** Overall, the governance landscape of Romanian public hospitals includes a large number of seemingly modern legislative provisions and management instruments. Over the past 30 years substantial efforts have been made to put in place standardised hospital classification, hospital governance structures, management and service purchasing contracts with key performance indicators, modern reimbursement mechanisms based on diagnosis-related groups (DRGs), and regulatory requirements for accountability, including internal and external audit. Nevertheless, their application appears to have been challenging for a range of reasons, pointing to the misalignment between the responsibility and decisionmaking capacity given to hospitals in a questionably conducive context. Incoherent policy design, outdated and often disjointed regulatory frameworks, and cumbersome administrative procedures limit managerial autonomy and obstruct efficiency gains. In a context of chronic insufficient funding, misaligned incentives, and overly rigid service procurement processes, hospitals seem to struggle to adjust service baskets to the population’s health needs or to overcoming financial hardship. External challenges, combined with the limited strategic, operational, and financial management capacity within hospitals, make it difficult to exhibit good financial and general performance.

**Conclusion:** Existing governance arrangements for Romanian public hospitals appear conducive to poor financial performance. The suggested framework for hospital governance assessment has proved a powerful tool for identifying system and hospital-specific challenges contributing to sub-optimal hospital performance.

## Background

Hospitals consume a substantial share of health resources worldwide: in Organization for Economic Cooperation and Development (OECD) member states, hospital expenditure represents between 26% and 53% of current health expenditure^1^ or between 1.5% and 5.7% of the national gross domestic product.^2^ The share of hospital expenditure from total health expenditure is comparable in developing countries – for example, 40% in Kenya,^3^ 29% in Tanzania,^4^ 36.5% in the Philippines,^5^ and 35% in India.^6^ At the same time, some international literature reports widespread overutilisation of hospital services as well as of wasteful use of hospital resources.^7^ Despite a relative focus on primary care in global health, hospitals maintain a central role, as attested, for instance, by the inclusion of access to “basic hospital services” in the service coverage monitoring framework for universal health coverage (UHC).^8^ As such, creating institutional capability for good hospital performance is an important issue, towards which governance arrangements play a vital role.

In Romania, public hospitals concentrate the majority of hospital infrastructure in terms of number of facilities (64%, n = 367 of 576 total hospitals) and of beds (94%, n = 131 337 beds of 140 339 total).^9^ Most public hospitals (80%) are run by local councils, with the remainder run by the Ministry of Health (MoH) or other ministries (eg, the Ministry of Transport, the Ministry of National Defence) or governmental institutions (eg, the Penitentiary Administration, the Romanian Academy). Hospital-related governance and financing policy changes occurred during the past decade, including: nearly 10% (~15 000) bed reduction of the oversized hospital infrastructure inherited from the communist period since 2005; a gradual reduction in hospital expenditure from 51% of the total public health expenses in 2005 to 37% in 2014 in the National Health Insurance Fund (NHIF)^10^; the transfer of hospital management from the MoH to local authorities; and the introduction of hospital accreditation and performance management agreements.^11^ Nevertheless, insufficient funding, suboptimal governance and inefficient use of resources persist in the Romanian hospital sector and health sector at large.^12,13^ Public hospitals’ accumulation of arrears – defined in the legislation as outstanding payments overdue by more than 90 calendar days – has long been an issue of concern both for the Government of Romania and its partners, including the European Commission and the World Bank.^14^

In 2015 the Government of Romania engaged international technical assistance to support the MoH in exploring the question “*Why do Romanian public hospitals accumulate arrears*?” Drawing insights from this work, we report here on an assessment of the governance arrangements of public hospitals with the underlying expectation that such an analysis would suggest key policy, technical and managerial interventions to improve public hospitals’ financial and overall performance.

## Methods

We structured our assessment using a public hospital governance framework with 4 dimensions^15^: institutional arrangements, financing arrangements, accountability arrangements and correspondence between responsibility and decision-making capacity (Table 1). Methodologically, the governance framework was operationalised using a 2-pronged approach: (*i*) a policy review of broader health system elements influencing hospital governance; and (*ii*) a series of 10 case-studies of public hospitals.

The policy review included key informant interviews, amongst them hospital managers, finance, accounting, human resources (HR), medical and Monitoring and Evaluation (M&E) directors, as well as MoH executives, representatives from the NHIF, National Authority for Quality Management in Healthcare (NAQMH), etc (list of interviewees in Supplementary file 1); and a document review of key health sector policies, regulations and norms defining hospital sector strategies, configuration, governance arrangements, financing and budgeting, service purchasing modalities, contracting mechanisms, monitoring and reporting, external audit, staffing, and quality assurance. For the case-studies, a purposive sample of 10 hospitals (of the existing 367 public hospitals in Romania) was selected based on pre-specified criteria agreed with the MoH: hospital classification as per the current legislation; geographic location (region, urban/rural); managing entity (MoH or local authorities); and history and magnitude of arrears. The objective of the sampling was to obtain as representative a sample as possible under all criteria, ie, a mix of managing entity, complexity and generalist-specialist hospitals, from at least 3 regions of Romania, with currently substantive arrears.

The activity of the sampled hospitals was documented using a mix of qualitative and quantitative methods. We developed a standardised data collection instrument based on the OECD healthcare quality indicators^16^ and on previous hospital performance measurements.^17,18^ The questionnaire comprised 150 questions structured in 5 blocks: (1) Hospital ownership, structure, and internal governance arrangements; (2) Hospital inputs (eg, physical assets, infrastructure, equipment and staff); (3) Hospital activity/processes (eg, population coverage, basket of services, clinical and managerial activity); (4) Hospital management, with a focus on financial management (costs and revenues, contracts, service purchasing mechanisms, outscoring, accounting, internal audit); and (5) Hospital outputs and outcomes (eg, quality of care, safety, patient satisfaction, efficiency and effectiveness). Additionally, a financial audit questionnaire was developed and applied by professional auditors compliant with ISAE 3000 standards.^19^ The Supplementary file 2 shows how questionnaire sub-sections map to each of the 4 domains of the governance framework in Table 1.

**Table 1:** Governance Framework for Public Hospitals

**Dimension**	**Key Questions**
Institutional	Who are you? What are your credentials? To what are you entitled? Are you recognized as “different and special,” or not?
Financing	What freedom do you have to handle your resources? From where do you get your money? How do you cope with your capital and revenue needs? What is your process for managing investments and running costs?
Accountability	On behalf of whom are you acting? To whom do you report? What kind of organisational structure do you have in that context? Who is involved in your decision-making processes?
Correspondence between responsibility and decision-making capacity	Can you honour your promises? Are you able to negotiate and reach agreements with others? How do you adjust to contingencies? How transparent are your day-to-day operations?

Data collection took place between September 2016 and March 2017 and entailed conducting semi-structured key informant interviews with hospital management teams and representatives of central authorities, and collecting hospital data using the standardised questionnaires through repeated site visits and face-to-face interviews with hospital management teams. The base year for hospital activity and financial data was 2015, with historical data for trends collected for the interval 2010-2015. The characteristics of the 10 sampled hospitals in relation to the selection criteria are summarised in Table 2 – obscuring for professional reasons the names of the hospitals.

**Table 2:** Characteristics of Hospitals Included in the Study

**Hospital**	**Geography (Development Region)**	**Ownership**	**Hospital Profile**	**Service Complexity Category (I–V)**	**Last Recorded Arrears**	**Magnitude of Last Recorded Arrears** ^a^
H1	North-East	Local authorities	Town	IV	November 2014	10.9%
H2	Bucharest	MoH	Emergency	I conformation plan	August 2016	<0.5%
H3	Bucharest	Local authorities	City mono-specialty	II M	December 2014	<0.5%
H4	West	Local authorities	Municipal	IV	August 2016	4.3%
H5	Bucharest	MoH	Institute	I M	July 2015	0.5%
H6	Bucharest	MoH	Emergency	II conformation plan	November 2013	n/a
H7	South	Local authorities	County	III	July 2016	<0.5%
H8	South	Local authorities	Municipal	IV	August 2016	33.2%
H9	South-West	Local authorities	County	III	May 2016	0.9%
H10	West	Local authorities	City mono-specialty	II M	August 2016	8.7%

Abbreviation: MoH, Ministry of Health. ^a^Calculated as a percentage of last recorded arrears relative to the period expenditure in the respective calendar year. For example, if a hospital last recorded arrears in October 2015, magnitude is calculated as % of arrears (October 2015) relative to the hospital’s budget execution January-October 2015.

## Results

In what follows, we present the findings of the assessment for the hospital governance framework’s 4 dimensions. For each dimension, we first outline the relevant regulatory framework and continue with findings from the case studies.

### Institutional Arrangements

Law 95/2006 (Title VII “Hospitals”) specifies the principles of classification, organisation and management of public hospitals. A number of formal hospital classifications are in place, based on ownership (public; public with private wards; and private), geography (regional; county; and local – municipal, city, or village), pathology (general; emergency; specialty; and chronic disease), medical research status (clinical hospitals and institutes) and clinical competencies (from category I, the most complex, to category IV, the least complex; category V includes specialist long-term care hospitals, such as mental health facilities).

The public hospital’s managing entity can be a legal or a physical person (“the manager”) entering a 3-year management contract (extended to 4 years in 2018) with the MoH or the local authority. The executive management of the hospital is ensured by a managerial board comprising the manager, the medical director, the financial director and, if the hospital has more than 400 beds, a care manager (ie, a nurse). The members of the managerial board are selected through contest and sign a 3-year performance agreement with the hospital manager, which includes performance indicators. Other internal structures mandated by Law 95/2006 are the medical council (with attributions towards monitoring and improving hospitals’ clinical activity performance), the ethical council (Art. 186, with specific attributions detailed in MO 145/2015), and the administration council (Art. 187). The administration council ensures the hospital strategic management, including but not limited to approving the budget and procurement plans and analysing the activity of the managerial board. Its exact composition is stipulated by Law, comprising 5 to 8 members, including representatives of the MoH or District Public Health Authorities (DPHA), representatives of the district or local council, a representative of the Mayor, representatives of the professional associations (Physicians’ Council and Nurses’ Council, both with ‘guest’ status) and, for teaching hospitals, a representative of the medical university. Separate legislation mandates the setup and functioning of other committees, such as the quality assurance committee and the haemovigilance and blood safety committee. Most committees have a consultative role and report to the management board; only the ethics committee is formally placed outside the hospital’s clinical governance arrangements and has broader decision-making powers, eg, it can independently alert the relevant public authorities in cases of reported bribery or misconduct.

All 10 hospitals in our study were able to produce a list of functioning committees and their respective operating procedures. However, there was considerable variation in the number of committees, from as little as 3, to as many as 45 (Table 3), and their membership (from one member to as high a number as 46), as well as the functions of the committees themselves. For example, some hospitals did not report having set up some committees mandated by law, while others reported setting up committees reflecting their specific needs, such as “the committee for declaring brain death” in H7; the “social dialogue committee” emphasising the relationship with the community (in H2 and H7), or with professional associations (H9). Several internal structures, including those with clinical remit, were dysfunctional at the time of the assessment – for example, the quality committee in H2, the infection control committee in H8 and the pharmacy inventory committee in H9 had no appointees.

**Table 3:** Internal Committees Reported by the Sampled Hospitals

**Hospital**	**No. of Internal Committees (of Which, Clinical Committees)**
H1	12 (6)
H2	11 (4)
H3	9 (4)
H4	3 (1)
H5	12 (5)
H6	12 (7)
H7	17 (9)
H8	5 (3)
H9	45 (10)
H10	18 (10)

Widespread distrust was noted among the hospital management teams towards the need for strategic leadership or governance arrangements. Hospital boards and committees were seen as cumbersome structures that obstruct decision making, as opposed to bringing added value. At least 2 managers admitted that “*people who are less busy are appointed on the committees.*” For example, H10:

“*The Board of Directors is strictly a formal committee. We get together and discuss things that we also discuss in the Medical Council. The Board of Directors approves all proposals, it is extremely open. While it does not impede the hospital’s activity, it does not facilitate it, either*.”

Regardless of typology and the official classification, all assessed hospitals face infrastructure-related challenges. The main buildings of sampled hospitals are dated from before the 1980s and are often scattered across multiple locations; 6 hospitals in the sample had buildings on more than one site (Table 4). There was little indication that procurement of medical equipment was informed by a situation analysis or business case which took into account the medical need among the target population, or the hospital’s own patient case-mix (ie, the measure of diversity, complexity and resource intensity of admitted cases). For example, a low complexity general hospital (level IV) in a small municipality possesses a computer tomography (CT) scanner.

**Table 4:** Selected Infrastructure and Equipment Availability in the Sampled Hospitals (2015 Data)

**Hospital**	**Complexity**	**Infrastructure**					**Equipment**					**HR**			
		**Year Main Building Built**	**No. of Sites**	**No. of Buildings (Units)**	**# Beds**	**No. of OT (OR)**	**X-Ray**	**CT**	**MRI**	**Laparoscopic**	**Radiotherapy**	**Total Actual Staff**	**Doctors**	**Nurses**	**Vacancies**
H1	IV	1977	4	4	193	1	Yes	Yes	No	Yes	No	244	36	89	33
H2	I	1967	1	6	725	6	Yes	Yes	Yes	Yes	Yes	2434	267^a^	848	420
H3	IIM	1894	1	5	119	3	Yes	Yes	No	Yes	No	195	11	88	71
H4	IV	1911	2	10	368	1 (4)	Yes	No	No	Yes	No	363	52	174	105
H5	IM	1906	5	13	632	3	Yes	No	No	No	No	711	90	270	98
H6	II	1972	1	4	525	17	Yes	Yes	No^b^		No	1776	166	425	314
H7	III	1974	5	7	1160	3	Yes	Yes	No	Yes	No	2164	168	942	337
H8	IV	1987	2	7	298	3	Yes	No	No		Yes	338	37	151	102
H9	III	1970	3	8	1153	1 (11)	Yes	Yes	Yes	Yes	No	1699	236	845	837
H10	IIM	1932; 1973^c^	1	11	325	1	Yes	No	No		No	389.5	43	114	14

Abbreviations: HR, human resources; OT, operating theatre; OR, operating room; CT, computed tomography; MRI, Magnetic resonance imaging. ^a^Additionally, there are more than 800 residents. ^b^Outsourced to a local private provider. ^c^The hospital has 2 main pavilions.

Likewise, diluted correspondence was observed between the basket of services rendered and the typology of hospitals. For example, the same Level IV general hospital provides cancer therapy alongside balneology services. Table 4 visualises unstructured patterns of defining and maintaining the infrastructure, equipment, and HR, with a little conjunction with the hospital profiles and the complexity of services.

Two of the assessed hospitals have a distinguished image in public, being considered the “reliable place to go” during medical emergencies. The search to understand the “success factors” resulted in emphases on the strong strategic leadership, stronger financial management and M&E systems and processes, top historical image, and the ability to attract the best medical cadre (largely due to a central location and existing image).

### Financial Arrangements

Law 95/2006 stipulates that public hospitals have “financial autonomy,” understood as the capacity to “*a) organize their activity based on the revenue and expenditure budget, approved by the management board and the respective credit release authority; and b) elaborate the budget based on evaluating revenues and expenditures informed by the proposals received from hospital wards and compartments*” (Art. 190). Different pieces of legislation govern the preparation and execution of budgets for hospitals administered by local authorities (Law 273/2006 on local finances) and by the MoH (MO 1043/2010 on budgeting for public hospitals). However, all public hospitals can receive funding from a wide range of sources: hospitals administered by the MoH can receive funding from state, county and local budgets as well as from the MoH, while hospitals administered by local authorities can receive funding from local budgets as well as from the state and MoH budget for medical equipment (with a contribution from local authorities of at least 10% of the project value), for major refurbishment (minimum 5% contribution), and modernisations/extensions (minimum 10% contribution).

In principle, revenues from the NHIF should cover the hospitals’ operating expenses with MoH funding only supporting capital investments. However, at the time of this assessment there were no formal criteria in place for allocating MoH funds, and substantial variations were observed in terms of the relative shares received from these sources (Table 5). Contracts with the NHIH represent the majority of funding, ranging from 41% to 86% of total revenues. The 3 MoH-administered hospitals received between 18% and 38% of revenue as subventions from the state budget, while hospitals administered by the LA did not receive any budget transfers.

**Table 5:** Revenue Sources for the Sampled Hospitals, as a Proportion of Total Revenues (%)

	**H1**	**H2**	**H3**	**H4**	**H5**	**H6**	**H7**	**H8**	**H9**	**H10**
Administered by	LA	MoH	LA	LA	MoH	MoH	LA	LA	LA	LA
Total	100%	100%	100%	100%	100%	100%	100%	100%	100%	100%
Property	0%	0%	0%	0%	0%	0%	0%	0%	0%	0%
Goods and services, of which	89%	43%	45%	87%	69%	56%	86%	91%	91%	99%
Contracts with health insurance houses	83%	42%	41%	82%	67%	56%	67%	86%	73%	56%
Capital and other financial operations	0%	0%	0%	4%	2%	0%	1%	0%	2%	0%
Subventions, of which	11%	57%	55%	9%	29%	43%	12%	9%	7%	1%
Direct from state budget	0%	29%	0%	0%	18%	38%	0%	0%	0%	0%
Direct from other administrations (including local councils)	11%	12%	55%	7%	3%	5%	11%	9%	7%	1%
From state budget via local councils, for investment	0%	0%	0%	2%	0%	0%	4%	0%	0%	0%
From MoH via local authorities, for investment	0%	0%	0%	0%	0%	0%	0%	0%	0%	0%

Abbreviations: LA, local authority; MoH, Ministry of Health.

In practice, funding arrangements was found to be more complex and opportunistic. For example, in one hospital (H9) the county council provided funding for food coupons, while the regional forensics institute covered the salaries of personnel involved in the hospital’s forensics service.

Year-on-year fluctuations in the hospitals’ net debt-surplus has been seen to rise up to (+/-) 20%-40% of their annual revenues (Table 6). One *Level IV* municipal hospital (H8) received cash inflow outside the normal course of operations from the municipality in order to cover outstanding liabilities – without it, the hospital would probably have incurred additional arrears. By contrast, a *level II* emergency hospital (H6) requests funds from the MoH one month in advance – should the MoH delay payments, this hospital would most probably incur arrears.

**Table 6:** Annual Debt/Surplus for the Sampled Hospitals, as a Proportion of Total Revenues (%)

**Hospital**	**2011**	**2012**	**2013**	**2014**	**2015**
H1	3.2%	0.9%	-0.2%	0.0%	2.5%
H2	-7.4%	-2.1%	4.5%	18.2%	25.0%
H3	5.5%	-12.2%	-31.1%	28.7%	45.6%
H4	4.7%	1.8%	-8.5%	-6.0%	-2.4%
H5	-0.8%	9.5%	-11.8%	7.2%	11.2%
H6	-8.1%	-2.1%	-4.6%	-5.2%	-3.8%
H7	-4.5%	-4.4%	-1.0%	6.3%	-3.1%
H8	-28.1%	-9.9%	-12.5%	49.2%	-88.9%
H9	14.1%	0.5%	1.6%	7.2%	-1.7%
H10	7.6%	-9.9%	-3.6%	6.6%	-5.4%

The main mechanisms of hospital funding comprise a mix of case-based payments from the NHIH, and lump sum payments for curative national health programmes (NHPs) from the MoH. Expenditure with NHPs was relatively constant between 2013 and 2016 at about RON 3 billion per annum (12%-14% of NHIF), while diagnosis-related group (DRG)-based reimbursements amount to RON 8-10 billion per annum.^10^ The Framework Contract with the NHIH is the main legislative tool regulating health service purchasing, issued every 2 years as a joint order signed by the Minister of Health and the NHIH President. The contract specifies in detail the benefits package (by service delivery level), provider payment mechanism, quality criteria, rights and obligations of the payer, providers and the insured etc. It also stipulates the formula for the total contracted sum (CS) for acute inpatient cases in DRG hospitals (equation 1), as well as the average length of stay (ALOS), Case Mix Index (CMI), standard bed utilisation rate and tariff for each hospital, based on historical activity in the previous year (eg, 2015 data informing the Framework Contract 2016-2017).

*CS = P X (number of contractible beds X bed utilisation rate/hospital average ALOS) X hospital Case Mix Index X tariff per adjusted case* (equation 1),

where *P* is an adjustment factor 62%-85% which depends on the hospital’s classification I-V.

While DRGs require up-to-date information on the average costs per type of case, the current DRG cost weights in Romania do not match the reality of hospital service costs, as long known and further confirmed by a 2012 review.^20^ The relative weights in current use are mainly drawn from the Australian weight structure, which was slightly altered in 2010 to include new definitions of cases and co-morbidities (but not significantly changed at its core). Moreover, the number of “contractible beds” for each hospital is decided annually by the respective county health insurance house based on the available funding (ie, county-level allocation from NHIF), and is usually lower than the total number of beds in the hospital structure.

All hospitals acknowledged that the contracting process with the county health insurance houses leaves almost no space for contract negotiations on price, service volumes and quality. Furthermore, county insurance houses apply invoice cut-offs, disregarding services actually delivered by hospitals in reporting periods when they exceed the agreed contract volume/value, expecting the hospital to cover the costs from other sources.

“*There is no actual negotiation with the [county] health insurance house. Money is divided based on the number of beds and per each medical unit. The amount established by the health insurance house is divided in quarters in the contract concluded with the hospital*” [H9].

“*There is no real negotiation between the hospital and the Health Insurance House*” [H3].

All hospitals also reported that the keeping of dozens of segmented programmes by the MoH is cumbersome from an administrative perspective (eg, each programme reimburses specific inputs) and represents a source of arguable decisions. Subsidies from local authorities are mandated by law, but in practice their quantum and frequency depend on the wealth of the concerned authority and introduce direct political influence in resource availability. Several hospitals commended the financial support of local authorities (eg, H9), while others (eg, H4 and H10) reported the need for more involvement in supporting the operating expenses of the hospital. In one case (H10), this would not be possible in the foreseeable future because* “the local council believes this will create a precedent for the other 2 hospitals it administers.”*

The majority of assessed hospitals questioned the need for a complete budget cycle of resource allocation, admitting that financial planning and budgeting are largely reduced to a simplified administration of revenues and expenditures. This was justified by acknowledging that “*nothing changes over the years, therefore there is no need to plan.*” Even the recommendations of negative financial audit reports lead to minimal changes. Two of the sampled hospital management teams admitted to deliberately incurring arrears as “*a means to attract attention of the Government or donors, and thus, additional funding.*” The financial audit identified instances where hospital management does not follow legislation and/or internal procedures concerning public acquisition, setting payroll-related taxes and addressing arrears.

### Accountability Arrangements

#### Performance Management

Hospital managers sign a contract with the managing entity (MoH or the local authority), which includes an annual performance assessment within the framework of 28 indicators grouped under 4 categories: HR (6 indicators), service utilisation (10 indicators), financial management (6 indicators), and quality of care (6 indicators). National average values for most of these performance indicators computed by the National School of Public Health and Management in 2007 (MO 1567/2007), by type of hospital, were used to set performance benchmarks. These average values have not been updated since and, in practice, have been mildly adjusted year-on-year, at the discretion of each oversight entity and so as to align with the actual hospital activity. According to the contract, the manager can be demoted if the performance indicators in the contract are not achieved for at least one year, and if that is the manager’s fault. In the initial hospital management contract (MO 1384/2010), the contract would cease (ie, the hospital manager would be automatically dismissed) due to incurring outstanding payments older than 2 years. In 2015, this interval was shortened to 3 months (MO 768/2015). For hospitals managed by the local authority, the hospital’s management board proposes the revocation of the manager.

The managers reported the absence of *de facto* support mechanisms when deviations from the target indicators arise, leading to inaccurate or, at best, mechanical reporting against the performance indicators towards matching the target values in the contract. For example:

*“Performance indicators are much too general and that they should be customised/adapted per hospital type *” [H10].

*“Performance indicators in hospital manager’s contract are NOT negotiated”* [H1].

“*Some of the indicators are relevant, some others not/some of them are or not under control – many of them are on medical activity and not on administrative activity as credit accountant*” [H7].

#### Information Management

MO 1571/2010 is the legislative foundation for a suite of medical services’ e-monitoring systems currently combined under the umbrella concept Health Insurance IT Platform (Platforma Informatica a Asigurarilor de Sanatate, PIAS). This comprises several systems, all administered by the NHIH. Relevant for hospitals are SIUI (Unique Integrated IT System), which records the detailed activity of health service providers for NHIF reimbursement purposes; CEAS (Electronic Health Insurance Card System), which monitors the use of health services by patients authenticated on the basis of the individual Health Insurance Card; and DES (Electronic Health Record), which records patients’ details and their full medical history. Additionally, all public hospitals report monthly to MoH on their financial activity, including debts and arrears. Data are analysed regularly by the MoH’s Integrity Department and monthly/quarterly reports are compiled. The MoH’s Audit Department also conducts regular audits in hospitals managed by the MoH. Cumulatively, these systems collect a large amount of information, yet there are no formal policy mechanisms to act upon their findings and the extent to which this information is used for policy decisions remains unknown.

Interviews with hospital managers and the analysis of activity data provided by the hospitals identified a range of data-related weaknesses, including: inconsistent reporting formats of in-patient activity across hospitals (eg, some report parallel sets of statistics for insured and uninsured patients, the same indicators reported either as rates or absolute number of cases from one hospital to another), arguable reliability and consistency regarding staff behaviour (especially patients’ complaints) and incomplete/inconsistent data collection for outcomes. Hospital managers acknowledged the weakness of M&E processes and practices, referring to fragmented monitoring, duplicate reporting to various stakeholders, lack of analytical capabilities and no formal incorporation of past lessons in future planning cycles.

#### Clinical Management

Quality of care is regulated in Romania at several levels, with the MoH, the NHIH and the National Authority for Quality Management in Healthcare (NAQMH) as key institutions. At highest level, Law 95/2006 stipulates that the MoH elaborates and implements measures for improving the efficiency and quality of health services (Art. 169, 1) and that the NHIH is responsible for ensuring the quality of health services comprised in the essential benefit package, based on criteria elaborated jointly with the MoH (Art. 238-239). Each hospital is mandated to elaborate/put in place its own clinical protocols, taking into account its specific context (eg, availability of equipment and staff). Quality management structures have been set up in hospitals (MO 975/2012), where the mandate includes implementing and monitoring the results of quality assurance mechanisms and training hospital staff in quality assurance. The second version of the NAQMH-elaborated hospital accreditation standards (MO 446/2017) entails reviewing the activity of each facility’s quality management structure in overseeing the existence, content and appropriate use of these protocols. There is no routine mechanism for monitoring the daily implementation of guidelines or the locally adapted protocols.

Interviews revealed the under-utilisation of quality assurance committees in improving clinical performance. Statistical information related to clinical outcomes was sparse – for example, few hospitals record systematic data on 30-day standardised mortality after admission for acute myocardial infarction (AMI) and ischaemic stroke, or the percentage of high risk transient ischaemic attack (TIA) patients treated within 24 hours of occurrence (Table 7). Moreover, many outcome indicator values appear either unrealistic (eg, 0 nosocomial infections) or display considerable year-on-year variation (eg, from 11.5% to 30.5% age-gender 30-day standardised mortality rate after admission for AMI). Despite all hospitals having at least some surgical activity (Table 8), most hospitals reported that causes of post-operative mortality are not regularly monitored, or could provide only an indicative list of causes unsupported by records. One hospital (H8) justified this situation: “*The hospital does not have post-operative mortality, because the surgeries performed are quite simple.*”

**Table 7:** Selected Quality of Care and Safety Indicators in the Sampled Hospitals

**Indicator**	**H1**	**H2**	**H3**	**H4**	**H5**	**H6**	**H7**	**H8**	**H9**	**H10**
Age-gender 30-day standardised mortality after admission for AMI (%)				11.5-30.5						
Age-gender 30-day standardised mortality after admission for AMI (cases)	0-2					4-31				
Age-gender 30-day standardised mortality after admission for stroke (%)				0.5-2.8						
Age-gender 30-day standardised mortality after admission for stroke (cases)	0					10-28				
Emergency readmissions within 28 days of discharge (%)	0	3.3-4.0		7.8-9.9	1.2-2.3				7.1-8.1	
Emergency readmission within 28 days of discharge (cases)						2041-2867				
Inpatient mortality rate	0.2-0.8	3.3-4.3		1.7-2.6	1.5-2	2.5-3.9	2.8-4.1		0.7-1.2	
Nosocomial infections (per 100 discharged patients)		0.8-1.0			0.05-0.5	0.1-0.2	0-0.7			0.1-0.3
Nosocomial infections (cases)	0-1			6					22-138	
Post-operative sepsis/complications (%)	0	1.8-2		0-0.07	0.15-0.45					
Pressure ulcers/bedsores (per 1000 beds)		3-28				78-99			38-66	
Unplanned returns to operating theatre (%)	0	1-2	0.7-1							

Abbreviation: AMI, acute myocardial infarction. Notes: blank spaces denote the respective indicators were not reported by the hospitals. Ranges are minimum-maximum annual averages over the 2011-2015 interval.

**Table 8:** Selected Output Indicators in the Sampled Hospitals (2015 Data)

	**No. of** **Doctors**	**No. of** **Beds**	**No.** **of OTs**	**ALOS (Days)**	**Average Occupancy Rate (%)**	**Surgical** **Interventions**	**Surgeries** **Per OT**	**Surgeries** **Per Doctor**	**Surgeries** **Per Nurse**
H1	36	193	3	11.3	90	1.139	380	32	13
H2	1315	725	6	6.4	86	25.254	4.209	19	30
H3	11	119	3	4.3	69	2.509	836	228	29
H4	52	368	4	8.0	60	2.661	665	51	15
H5	90	632	3	14.2	91	3.368	1.123	37	12
H6	166	525	17	6.3	81	10.255	603	62	20
H7	168	1.160	3	7.0	80	25.997	8.666	155	28
H8	37	298	3	5.5	49	1.416	472	38	9
H9	236	1.153	11	7.6	78	1.639	149	7	2
H10	42	315	1	11.0	70	179	179	4.3	1.6

Abbreviations: ALOS, average length of stay; OT, operating theatre.

### Decision-Making Capacity Versus Responsibility

One of the most remarkable findings of the study is that while legislative provisions confer autonomy to hospital managers over a wide range of managerial decisions, including financial management, HR and hospital structure, hospital managers systematically reported having “*almost zero managerial autonomy.*” Managers acknowledged limited authority to change their hospital’s organisational structure (eg, structure of wards, number of beds and staff) without the approval of the MoH or the local authority. Changing the ward structure could only be obtained through lengthy procedures (taking up to 6 months for MoH-administered hospitals) and would also bring upon the hospital the obligation to undergo a fresh accreditation cycle, with associated costs (thus discouraging managers from pursuing such reorganisations). Hiring personnel for already approved positions in the organogram also requires the MoH or local authority’s approval, etc.

Funding insufficiency was identified by all managers as the key challenge to their operations and was partly linked to the allocation and utilisation of funds contracted from the county health insurance house. For example:

“*With regards to arrears, the amount needed for their coverage is approximately equal to the amount not paid by the health insurance house (corresponding to medical services delivered in excess of the contract threshold). If the house reimbursed the medical services delivered and not only the services contracted, this hospital would be able to pay arrears, or even achieve a surplus”* [H10].

*“The district health insurance house contracts approximately 88% of medical services delivered by the hospital, but with significant variations, between 60% - 98%. The list of reasons for debt accumulation is supplemented by contracting rules and formulas, discriminating measures that do not allow the conclusion of contracts that consider the true capacity and needs of the hospital. This in turn leads to a distorted funding practice that is completely unrelated to the real and actual costs of our hospital, of many services, pathologies/specialties, and to the obsolete infrastructure that generates operational expenditures above average”* [H4].

Most hospital managers mentioned their responsibility of admitting any patient who would come through the hospital’s door, irrespective of insured status. The only reason why hospitalisation would be refused would be “*cases that cannot be treated in our hospital due to equipment shortage or inexistent specialty*” (H9). A number of hospital managers (eg, H6 and H10) invoked the “*social mission of the hospital*”, which usually comes to the fore during winter months, when hospital management confronts a sharp increase in “*social cases.*” Most of these are homeless patients who have nowhere to go and remain admitted for much longer periods than warranted by medical need, if there ever was any. Their volume was informally estimated by some managers (eg, H9) at 10% of total cases at a given point in time and was linked to the absence of social care centres in their localities. This situation, in their opinion, strains the hospital’s budget because some cases are not emergencies (which are reimbursed separately from DRGs) and patients are often uninsured, (therefore the inpatient episode cannot be reimbursed from the NHIF and hospitals need to cover the associated costs from other sources).

Similarly, a perceived “social” responsibility on maintaining hospital jobs seems to have a strong influence on the managers’ operational decisions. For example, H9 considered the decision to outsource laundry services but eventually declined to do so because the external laundry company would have taken over the entire personnel and jobs would have been lost (personnel in the hospital’s food unit were requalified for laundry services, at the hospital’s expense, to keep laundry function internal).

There are also issues of organisational and professional culture. Legislation stipulates a bottom-up approach to budgeting, with chief of departments/wards drafting annual budgets and submitting them to the hospital manager for consolidation. In cases where the manager does not have a clinical background, this exercise can become mechanistic.

“*As an economist, the manager of a hospital does NOT have the competencies to correct/challenge the doctors’ budget”* [H7].

## Discussion

### Summary of Findings

The governance arrangements of Romanian hospitals include a large number of seemingly modern legislative provisions, regulatory frameworks and management instruments, such as competency-based hospital classification, reimbursement using DRGs, hospital boards, and management performance contracts with standardised performance indicators. Their application, however, appears problematic for a wide range of reasons. There are instances of incoherent policy design and misaligned or contradictory incentives – for example, DRG-based reimbursement is used by the NHIH in the direction of gradually reducing the number of hospital beds, but hospital staffing norms are linked directly to the number of beds, making managers feel pressured to avoid layoffs. In other cases, incomplete regulatory frameworks undermine the entire purpose of the respective policy – for example, lack of updated target values for standardised performance indicators makes most performance contracts a formality whereby target values and indicator reporting become meaningless. Furthermore, the disconnect between DRG tariffs and the real cost structure faced by hospitals makes NHIH reimbursement an unreliable basis for financial planning because the recovery of operational costs is often not even feasible. In addition to the financing and service purchasing limitations, formally declared managerial autonomy is eroded by the cumbersome administrative procedures (eg, changing the organisational structure of the hospital). This limits the managers’ decision-making capability and contributes to perpetuating the status quo, ie, maintaining a large number of vacant positions.

The core challenges identified by the assessment in the 10 sampled hospitals are summarised in Table 9. Some challenges were identified in all hospitals, such as insufficient funding, limited managerial autonomy and meaningless contract negotiations with the insurance house. There was little indication of the hospital management’s ability to overcome those challenges and despite formal statements to the contrary, such elements appear as immutable components of the context.

**Table 9:** Synthesis of Governance-Related Challenges Identified in Sampled Hospitals

**Institutional Arrangements**	**Accountability Arrangements**	**Financing Arrangements**
• Underappreciation for the role of strategic leadership in hospital management • Dysfunctional governance bodies/committees • Resource heavy (outdated and spread over) infrastructure Un-systematic equipment planning and procurement	• Rigid performance framework • Uneven, incomplete systems and processes for information management • Challenges with clinical management	• Insufficient funding • Complex, burdensome funding streams • Rigid DRG-based contracting and reimbursement • Poor financial management, procurement and outsourcing • Poor or absent internal auditing and poor response to external audit
**Correspondence between Responsibility and Decision-Making Capacity**		
• Zero managerial autonomy • Social mission of the hospital towards the community ~ unnecessarily long stays, keeping jobs • Regulations and governance arrangements forcing to maintain unnecessary and costly infrastructure and vacant posts		

Abbreviation: DRG, diagnosis-related group.

Other challenges were reported or identified in some of the hospitals, but not in all of them – for example, single speciality hospitals raised more acutely the issue of overly rigid and generic performance indicators, which do not reflect their particular case-mix and service delivery structure. In a similar vein, some hospitals reported receiving consistent financial support from the local authorities, while others reported obstacles in engaging local stakeholders to contribute towards hospital operations.

As a consequence, there was substantial variability in the sampled hospitals’ material and staff endowment, service production and practical implementation of governance arrangements. Some, but not all of this variability, can be explained by the hospital complexity level – more complex hospitals (level I and II) in the sample were generally better endowed and had more comprehensive monitoring processes and systems. For most hospitals however, the link between endowment, service organisation and the medical need, or burden of disease in their respective catchment area is unclear.

### Analysis of Findings

Figure synthesises the hospital governance related challenges by the assessment framework pillars. On the revenue side, hospitals are funded through complex arrangements that involve multiple stakeholders and contracting frameworks. There is a distinct focus on top-down cost control: the majority of hospital funding comes from the NHIH, which is determined in practice by historical allocations and the year-on-year funding availability. Table 5 highlights the variability of revenues from the MoH and the NHIH by hospitals. Moreover, the NHIH funding formula includes multiple conflicting parameters, eg, number of contractible beds and also case-mix index, leaving very little (if any) space to address the ever-changing service delivery needs and respective estimation of hospital revenues. In fact, it allows the complete alternation of the true DRG design and virtually eliminates any opportunity for hospital managers to use the DRG-based payments for efficiency and effectiveness improvements.

**Figure:**
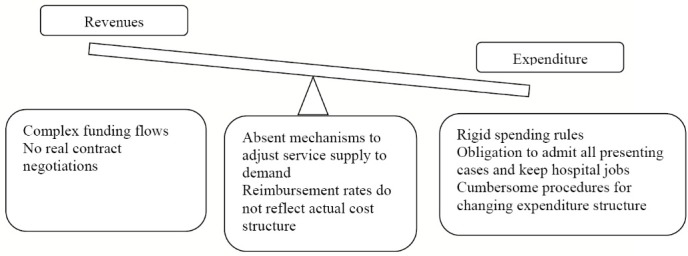
Influence of Governance Arrangements on Financial Performance in Romanian Public Hospitals.

On the expenditure side, there are no transparent mechanisms to regulate the demand for services (eg, *de facto* admittance of nearly every case and “*social mission*”) or to adjust funding in response to demand (eg, no real negotiations with the insurance house); and managerial autonomy is hampered by contradictory legislative provisions (eg, the link between beds, revenues and staffing) and cumbersome procedures (eg, structural reorganisation). In such an environment, the accumulation of arrears in Romanian public hospitals has lost much of its meaning as a marker of sub-optimal hospital performance.

This study identifies the core challenges of the inability to address the substantial inconsistency in the endowment, service production and outcomes across the sampled hospitals. It is unclear for most facilities how outputs relate to the burden of disease; duplications as well as inefficiencies are also widespread, with facilities of different levels essentially producing much of the same type of services. Most business processes are not rigorously designed, are applied rather haphazardly and are insufficiently monitored. It is also unclear how quality of services can be improved either by the hospitals themselves (with current internal quality assurance mechanisms) or by inspections, accreditation, and comparison with other hospitals.

The response (or lack thereof) by hospital managers to the constraints in organising and running their facilities also points to insufficient strategic, operational, and financial management capabilities. Inconsistent decision-making in hospitals, sub-optimal quality of care and insufficient patient-centeredness are much more serious challenges than arrears themselves. In this sense, it can be said that existing governance arrangements for public hospitals in Romania appear conducive to poor financial performance and stimulate complex relationships between the policy maker, purchaser and service provider.

Discussions with project stakeholders revealed that most of the identified challenges have been well-known for years – for example, the limitations of the current DRG system have long been documented.^21^ This only reveals the deep nature of the issues analysed in this paper. Historically speaking, poor strategic planning and unclear business models appear to have caused misalignment between hospital capacity and health needs, and between baskets of services and category of facilities in Romania. Hospital sector policies over many years, enacted through conflicting or outdated regulatory frameworks, have incentivised hospitals to maintain excessive, inefficient infrastructure and weak accountability mechanisms. The challenge for going forward is to find a consensus-based mechanism to solve those imbalances.

### Strengths and Limitations of the Study

The main strength of our study derives from using a structured and comprehensive governance assessment framework, designed for public hospitals and supported by ample data collection at central and hospital level. We believe our findings offer a broad, in-depth view of how Romanian public hospitals really function.

There are also limitations. While every effort was made to include a representative sample of hospitals, the cohort of 10 hospitals, chosen by the MoH, is hardly representative in a statistical sense. Also, the absence of a control group (hospitals with no history of arrears) makes it difficult to ascertain the specific factors which determine the accumulation of arrears and, more broadly, poor hospital performance. Some data requested from hospitals on their clinical and financial activity could not be provided in time for the analysis – the availability of time series for outcome indicators was particularly problematic.

Also, not all governance issues suggested in the Framework/summarised in Table 1 were covered in the same depths.

### Relationship With Other Studies

To our knowledge, this is the first in-depth, systematic assessment of public hospitals in Romania and one of the first assessments of governance arrangements for public hospitals in Eastern Europe. Previous in-depth service delivery assessments in Romania focused on primary care services.^22^ Some of our findings are aligned with previous studies – a recent analysis identified price reduction as the key criterion driving drug policy decisions in Romania.^23^

Other studies of factors affecting decision-making processes in public hospitals had similar findings. For example, the factors that affect priority setting and resource allocation decision practices, as identified in a recent case-study of 2 Kenyan public hospitals, were inadequate financing level and poorly designed financing arrangements; limited hospital autonomy and decision space; and inadequate management and leadership capacity in the hospital.^24^ Systematic attempts at characterising hospital governance in low and middle-income countries focussed on specific topics, such as autonomy.^25^

### Implications for Practice

The analysis informed a set of recommendations that were put forward to the Romanian MoH in 2017. These focused on setting a consensus-based policy process to review and reform the hospital sector configuration and governance arrangements. This process would entail improving hospital sector capacity planning, revisiting hospital classification and standards; revising hospital governance structures and oversight mechanisms; improving hospital financing and service purchasing arrangements. Recommendations on institutional improvement included reconsidering the business models and models of care within hospitals, based on the population’s health needs and realistic acknowledgment of financial limitations; investing in financial management and internal audit systems and processes; and putting emphases on continuous capacity building with a focus on advanced managerial competences.

## Conclusion

The assessment of governance arrangements in a sample of Romanian public hospitals proved a powerful tool for identifying system and hospital-specific challenges contributing to poor financial and overall performance, which have been documented. Similar assessments conducted elsewhere may constructively have impact in diagnosing hospital and health system performance, and formulating policy interventions for improved hospital service organisation and delivery.

## Acknowledgements

The work was undertaken by Oxford Policy Management and Ernst & Young Romania. We want to acknowledge valuable inputs by EY in financial audit as well as data collection for general performance assessment. Oxford Policy Management led the analyses and reporting.

## Ethical issues

Permission for data cllection and publishing was received from the MoH Romania.

## Competing interests

Authors declare that they have no competing interests.

## Authors’ contributions

TC and AD designed the study. AD, AM, AG, and TC analysed the data. TC and AG wrote the draft. All authors revising the manuscript critically for important intellectual content. All authors have read and approved the final version of the manuscript and approve of its submission to this journal.

## Funding

This work was supported by the World Bank.

## Authors’ affiliations

^1^ALLDMHEALTH, Seville, Spain. ^2^Oxford Policy Management, Oxford, UK.

## Supplementary files

Supplementary 1. List of interviewees.

Supplementary 2. Alignment between the Framework dimensions and Questionnaire structure.

## Key messages

**Implications for policy makers**

- Existing governance arrangements for public hospitals in Romania are linked to poor financial performance. On the revenue side, hospitals are funded through rather contradictory arrangements and in practice the majority of hospital funding is determined by historical allocations and year-on-year funding availability. On the expenditure side, there are no mechanisms to regulate or respond to the demand for services. In addition, managerial autonomy is limited by contradictory legislative provisions.
- From a historical perspective, poor strategic planning and unclear business models have caused misalignment between hospital capacity and health needs, and between baskets of services and category of facilities.
- Much needed reform of Romania’s hospital sector would entail: revisiting hospital classification and standards; revising hospital governance structures and oversight mechanisms; reconsidering the business models and models of care within hospitals (based on the population’s health needs); emphasising continuous capacity building with focus on advanced managerial competences; and improving hospital financing and service purchasing arrangements.

**Implications for public**

Hospitals represent a significant proportion of health spending in many countries but the determinants of their performance (and lack thereof) are often complex and difficult to discern in practice. We present lessons drawn from a comprehensive governance assessment of 10 public hospitals in Romania, which include document review, key informant interviews and in-depth data collection using a standardised questionnaire. Such an assessment proves a powerful tool for identifying system and hospital-specific challenges contributing to poor performance, and calls for policy interventions towards improved hospital service organisation and delivery with increased patient involvement.
