# Supplementary material for: Assessment of Public Hospital Governance in Romania: Lessons From 10 Case Studies
Source: Int J Health Policy Manag. 2018 Dec 22;8(4):199–210. doi: 10.15171/ijhpm.2018.120 (PMC6499904; doi:10.15171/ijhpm.2018.120)
Supplement: Supplementary file 1 — Supplementary 1. List of interviewees. [file ijhpm-8-199-s001.pdf]

Supplementary 1. List of interviewees

Organisation	Representative(s)
Central level	
Ministry of Health	State secretary in charge of the project<br>Various departments: Legal; Budget; Audit; Integrity
National Health Insurance House	Hospital contracting team
National Authority for Health Management and Quality	Managing Director
National School of Public Health, Management and Professional Development Bucharest	Executive Director; Deputy Director.
Case-study hospitals	
H1	Hospital manager; Quality manager; HR manager; Prevention of nosocomial infections lead; IT consultant; Building administrator.
H2	Hospital manager; Finance and accounting director; statistician; HR director.
H3	Hospital manager; Medical director; Finance and accounting director; HR director; Head of Administrative Services.
H4	Hospital manager; Medical director; Finance and accounting director; Building administrator.
H5	Hospital manager; Medical director; Finance and accounting director; Statistician.
H6	Hospital manager; Medical director; Finance and accounting director; Statistician; HR director; Legal adviser; Head of technical office.
H7	Hospital manager; Finance and accounting director.
H8	Hospital manager; Medical director; Finance and accounting director; Head of surgical department; Statistician; HR director; Legal adviser; Head of technical office.
H9	Hospital manager; Medical director; Finance and accounting director; HR director; Head of procurement.
H10	Hospital Manager; Finance and Accounting director; Medical director; HR director; Statistician; Legal adviser; Head of technical office; Head of service quality management; Head of procurement.
