# Supplementary material for: Assessment of Public Hospital Governance in Romania: Lessons From 10 Case Studies
Source: Int J Health Policy Manag. 2018 Dec 22;8(4):199–210. doi: 10.15171/ijhpm.2018.120 (PMC6499904; doi:10.15171/ijhpm.2018.120)
Supplement: Supplementary file 2 — Supplementary 2. Alignment between the Framework dimensions and Questionnaire structure. [file ijhpm-8-199-s002.pdf]

Supplementary 2. Alignment between the Framework dimensions and Questionnaire Structure

Framework Dimension	Key questions	Questionnaire sections
Institutional	Who are you?<br>What are your credentials?<br>To what are you entitled?<br>Are you recognized as "different and special", or not?	Hospital name, applicable category/levels of care<br>Characteristics of the catchment population<br>Organisational Chart<br>Internal governance structures and processes
Financing	What freedom do you have to handle your resources?<br>From where do you get your money?<br>How do you cope with your capital and revenue needs?<br>What is your process for managing investments and running costs?	Breakdown of buildings, high performance equipment, staff<br>Fixed assets, inventories, trade and other receivables, trade and other payables<br>Time series of revenues and expenditure, by source and destination<br>Contracting processes, instruments and templates<br>Resource allocation rules (formal and informal)<br>Internal auditing
Accountability	On behalf of whom are you acting?<br>To whom do you report?<br>What kind of organizational structure do you have in that context? Who is involved in your decision-making processes?	Legal status, ownership, administrative entities<br>External governance structures and processes, including accountability requirements, reporting, external auditing<br>Customer services and patient rights<br>Measuring patient satisfaction
Correspondence between responsibility and decision-making capacity	Can you honour your promises?<br>Are you able to negotiate and reach agreements with others?<br>How do you adjust to contingencies?<br>How transparent are your day-to-day operations?	Open comments on the relationship between the 3 dimensions above – institutional, financing and accountability
